# Differential Prognostic Impact of IABP-SHOCK II Scores According to Treatment Strategy in Cardiogenic Shock Complicating Acute Coronary Syndrome: From the RESCUE Registry

**DOI:** 10.3390/medicina60010183

**Published:** 2024-01-20

**Authors:** Bum Sung Kim, Woo Jin Jang, Ki Hong Choi, Sung Hea Kim, Cheol Woong Yu, Jin-Ok Jeong, Hyun Jong Lee, Hyeon-Cheol Gwon, Hyun-Joong Kim, Jeong Hoon Yang

**Affiliations:** 1 Division of Cardiology, Department of Medicine, Konkuk University Medical Center, Seoul 05030, Republic of Korea; dolphindance98@gmail.com (B.S.K.); shkim@kuh.ac.kr (S.H.K.); 2 Department of Cardiology, Ewha Woman’s University Seoul Hospital, Ehwa Woman’s University School of Medicine, Seoul 07804, Republic of Korea; wj78914@gmail.com; 3 Division of Cardiology, Department of Medicine, Heart Vascular Stroke Institute, Samsung Medical Center, Sungkyunkwan University School of Medicine, Seoul 06351, Republic of Korea; cardiokh@gmail.com (K.H.C.); hcgwon@naver.com (H.-C.G.); 4 Division of Cardiology, Department of Internal Medicine, Korea University Anam Hospital, Seoul 02841, Republic of Korea; ycw717@naver.com; 5 Division of Cardiology, Department of Internal Medicine, Chungnam National University Hospital, Daejeon 35015, Republic of Korea; jojeong@cnu.ac.kr; 6 Division of Cardiology, Department of Medicine, Sejong General Hospital, Bucheon 14754, Republic of Korea

**Keywords:** cardiogenic shock, acute coronary syndrome, intra-aortic balloon pumping, extracorporeal membrane oxygenation

## Abstract

*Background*: Early risk stratification is necessary for optimal determination of the treatment strategy in cardiogenic shock (CS) complicating acute coronary syndrome (ACS). Therefore, we evaluated the prognostic impact of an intra-aortic balloon pump on the cardiogenic shock (IABP-SHOCK) II score according to the treatment strategies in ACS complicated by CS using the RESCUE (REtrospective and prospective observational Study to investigate Clinical oUtcomes and Efficacy of left ventricular assist device for Korean patients with cardiogenic shock) registry. *Methods*: The RESCUE registry contains multicenter observational retrospective and prospective cohorts that include 1247 patients with CS from 12 centers in Korea. A total of 865 patients with ACS complicated by CS were selected and stratified into low-, intermediate- and high-risk categories according to their IABP-SHOCK II scores and then according to treatment: non-mechanical support, IABP, and extracorporeal membrane oxygenators (ECMOs). The primary outcome was all-cause mortality during follow-up. *Results*: The observed mortality rates for the low-, intermediate-, and high-IABP-SHOCK II score risk categories were 28.8%, 52.4%, and 69.8%, respectively (*p* < 0.01). Patients in the non-mechanical support and IABP groups showed an increasingly elevated risk of all-cause mortality as their risk scores increased from low to high. In the ECMO group, the risk of all-cause mortality did not differ between the intermediate- and high-risk categories (HR = 1.21, 95% CI: 0.81–1.81, *p* = 0.33). The IABP-SHOCK II scores for the non-mechanical support and IABP groups showed a better predictive performance (area under curve [AUC] = 0.70, 95% CI: 0.65–0.76) for mortality compared with the EMCO group (AUC = 0.61, 95% CI 0.54–0.67; *p*-value for comparison = 0.02). *Conclusions*: Risk stratification using the IABP-SHOCK II score is useful for predicting mortality in ACS complicated by CS when patients are treated with non-mechanical support or IABP. However, its prognostic value may be unsatisfactory in severe cases where patients require ECMOs.

## 1. Introduction

Cardiogenic shock (CS) is a circulatory failure event associated with an insufficient oxygen supply to various tissues that is mainly driven by low cardiac output [1,2]. Acute coronary syndrome (ACS) was reported to be the most common cause of CS [3,4]. Currently, early reperfusion with primary percutaneous coronary intervention (PCI) is the first-line management strategy to improve the clinical outcomes in patients with ACS complicated by CS [5,6], and advances in medical management and mechanical circulatory support devices have been used in clinical practice [7]. Despite many therapeutic advances over the past two decades, the mortality rate of ACS-complicated CS still approaches 30–50% [8,9]. Accordingly, early recognition and initiation of CS management is essential, and risk stratification is necessary for determining the optimal treatment strategy. However, treatment strategy selection is challenging in cases with heterogeneous conditions complicated by CS [10]. The previously published intra-aortic balloon pump in cardiogenic shock II (IABP-SHOCK II) risk scores, based on the IABP-SHOCK II trial, are easily calculated in daily clinical practice and strongly correlated with mortality in patients with infarct-related CS [11]. Nevertheless, because CS patients using advanced mechanical circulatory support (MCS) devices, such as extracorporeal membrane oxygenators (ECMOs), were not included in the IABP-SHOCK II trial, the IABP-SHOCK II score provides limited information for patients with ACS complicated by CS who are receiving advanced MCS [12]. Therefore, we investigated whether the prognostic value of the IABP-SHOCK II scores are different in ACS cases complicated by CS according to the treatment strategy (non-mechanical support, IABP, and ECMOs) using data from a dedicated multicenter CS registry.

## 2. Materials and Methods

### 2.1. Study Design and Study Population

**Figure 1:**
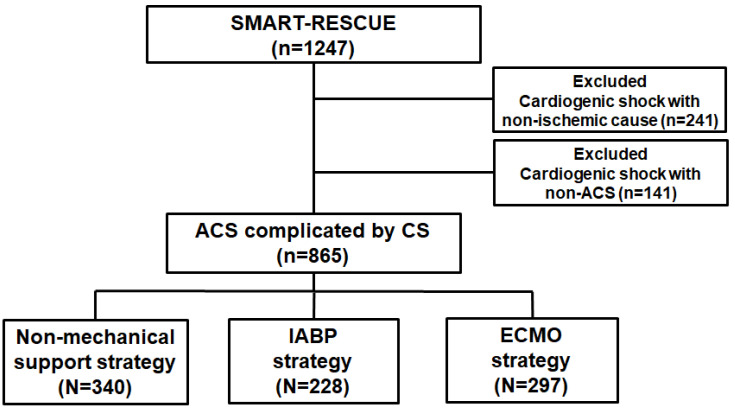
Schema of the study population distribution.

The RESCUE (REtrospective and prospective observational Study to investigate Clinical oUtcomes and Efficacy of left ventricular assist device for Korean patients with cardiogenic shock, http://www.clinicaltrials.gov/study/ NCT02985008, accessed on 19 January 2024) study is a multicenter retrospective and prospective registry of patients with CS [13]. Between January 2014 and December 2018, a total of 1247 CS patients (954 were enrolled retrospectively and 293 prospectively) were recruited from 12 tertiary centers. The inclusion criteria were (1) being ≥19 years old, (2) having a systolic blood pressure < 90 mmHg for 30 min or a state that required inotrope or vasopressor support to achieve a systolic blood pressure > 90 mmHg, and (3) the presence of pulmonary congestion and signs of impaired organ perfusion (altered mental status, cold skin, urine output < 0.5 mL/kg/h for the previous 6 h, or blood lactate > 2.0 mmol/L). The major exclusion criteria were out-of-hospital cardiac arrest, other causes of shock than CS (hypovolemic or septic shock), and patient refusal of active treatment. From this registry, we selected data from 865 patients who presented with ACS complicated by CS for analysis (Figure 1). The study protocol was approved by the Institutional Review Board (IRB) of each study hospital and was conducted according to the ethical guidelines of the 2013 Declaration of Helsinki. All IRBs waived the requirement for informed consent for retrospectively enrolled patients, and all prospectively enrolled patients provided written informed consent before enrollment.

### 2.2. Data Collection, Outcomes, and Risk-Scoring System

The RESCUE registry information was collected by independent clinical research coordinators via web-based case-report forms, including patient demographics, in-hospital management, laboratory data, procedural data, and outcomes. Additional information was obtained by further inquiring into medical records or making telephone contact, if necessary. The primary outcome was all-cause mortality during follow-up. A risk-scoring system was adopted using the IABP-SHOCK II score [11]. In this study, successful revascularization was defined as a TIMI flow grade of 3 after PCI was achieved or coronary bypass surgery was performed. According to the IABP-SHOCK II score, the population was classified into three risk categories: low (0–2), intermediate (3–4), and high (5–9).

### 2.3. Procedures

Coronary interventions and the best available medical treatments were provided in accordance with the standard guidelines at the time of each procedure [5,14]. The revascularization strategy was selected at the operator’s discretion. All patients received loading doses of aspirin (300 mg) and P2Y12 inhibitors (clopidogrel 300–600 mg, ticagrelor 180 mg, or prasugrel 60 mg) before PCI unless the patient had previously received these medications. To maintain the optimal organ perfusion during shock management, the choice of inotrope or vasopressor initiation, type, dose, and combination, as well as target blood pressure, were determined according to the operator’s discretion. MCS devices were initiated when the CS patients were unresponsive to vasopressors after correction for hypovolemia and hypoxemia or when arrest was prolonged or recurrent. The decision to implant an ECMO, an IABP, or both was made by experienced interventional cardiologists or cardiac surgeons. ECMO devices were inserted via percutaneous cannulation using the Seldinger technique or surgical cannulation using the cut-down method at the femoral vessels. In the event of distal limb ischemia after arterial cannulation, a catheter was inserted distal to the cannulation site for limb perfusion. IABPs were inserted percutaneously through the femoral artery with fluoroscopy guidance. Patients receiving concomitant ECMOs and IABPs were classified into the ECMO group.

### 2.4. Statistical Analyses

Comparisons of continuous variables were made using ANOVA or Kruskal–Wallis tests as applicable, and the results are presented as means ± standard deviations. Continuous variables were transformed into categorical variables and were assessed using the cut-off values determined in a reference study [11]. Categorical variables were evaluated using the chi-square test or Fisher’s exact test with Bonferroni correction for multiple testing as appropriate, and the results are presented as numbers and relative frequencies. Survival curves were constructed using Kaplan–Meier estimates and compared using the log-rank test. The Cox proportional hazard model was used to compare the risks of all-cause mortality between each of the risk categories and CS management strategies. The discrimination performance of the IABP-SHOCK II score for all-cause mortality according to treatment strategy (non-mechanical support/IABP vs. ECMO) was evaluated using a two-tailed paired comparison of ROC analyses (DeLong’s method). All probability values were two or three-tailed, and *p*-values < 0.05 or <0.017 were considered statistically significant. Statistical analyses were performed using SPSS 20.0 (SAS Institute Inc., Cary, NC, USA) and the R statistical software (version 3.6.0; The R Foundation for Statistical Computing, Vienna, Austria).

## 3. Results

### 3.1. Baseline Characteristics

Among the 1247 registered patients, 865 presented with ACS complicated by CS. We classified the 865 patients into three groups according to the CS management strategy: 340 patients (39.3%) were in the non-mechanical support group, 228 (26.4%) were in the IABP group, and 297 (34.3%) were in the ECMO group. Their baseline characteristics are shown in Table 1. The mean age of the total study population was 67.4 ± 12.4 years and 630 patients (72.8%) were men. A total of 552 patients (63.8%) presented with ST-segment elevation myocardial infarction. Compared with patients in the non-mechanical support group, those in the IABP and ECMO groups had a higher prevalence of diabetes and previous myocardial infarction. Additionally, the patients in the IABP and ECMO groups had higher creatinine, glucose, and lactate levels. The patients in the IABP group were older compared with those in the non-mechanical support or ECMO groups. The angiographic findings and in-hospital management are presented in Table 2. Patients treated with an IABP or ECMO had multi-vessel coronary artery disease, left main or proximal left anterior descending artery involvement, or unsuccessful or unexecuted revascularization and were likely to receive higher numbers of inotropes or vasopressors, a mechanical ventilator, and continuous renal replacement therapy.

### 3.2. Overall Mortality According to the IABP-SHOCK II Score

**Figure 2:**
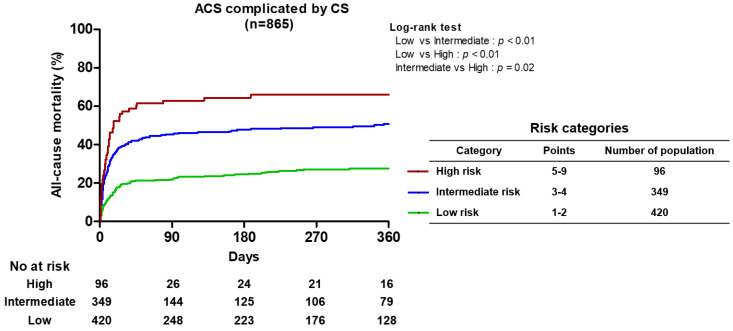
Kaplan–Meier curve for all-cause mortality according to IABP-SHOCK II score.

Overall, 371 deaths (42.9%) were observed among our subjects. Of these, 308 (35.6%) were in-hospital deaths, and 63 (7.3%) were post-discharge deaths during the median follow-up duration of 335 days (IQR 73–376 days). Using the IABP-SHOCK II score, 865 patients were classified into three risk score categories (low, intermediate, and high): 420 (48.5%) were at low risk, 349 (40.3%) were at intermediate risk, and 96 (11.2%) were at high risk. For each risk category of low, intermediate, and high, the observed mortality rates were 28.8%, 52.4%, and 69.8%, respectively (*p* < 0.01; Figure 2, Table 3).

### 3.3. Mortality According to the IABP-SHOCK II Score and Management Strategy

**Figure 3:**
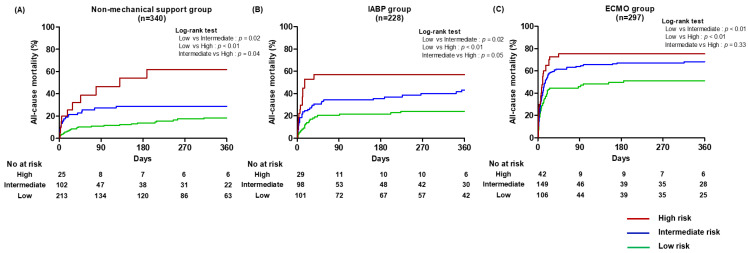
Kaplan–Meier curve for all-cause mortality according to IABP-SHOCK II score in the non-mechanical support (**A**), IABP (**B**), and ECMO (**C**) groups.

For each management strategy (non-mechanical support, IABP, and ECMO), the observed overall mortality rates were 25.3%, 37.7%, and 67.0%, respectively (*p* < 0.01). Table 3 and Figure 3 show the all-cause mortality for each management strategy according to the IABP-SHOCK II score risk category. In the non-mechanical support group, intermediate-score (hazard ratio [HR] = 2.06, 95% confidence interval [CI]: 1.27–3.32, *p* < 0.01) and high-score category patients (HR = 5.33, 95% CI: 2.91–9.75, *p* < 0.01) showed a significantly higher risk of all-cause mortality during follow-up than the low-score category patients. The high-score category patients showed a significantly higher risk of all-cause mortality compared with the intermediate-score category patients (HR = 2.39, 95% CI: 1.31–4.36, *p* < 0.01). In the IABP group, the intermediate-score (HR = 2.05, 95% CI: 1.24–3.36, *p* < 0.01) and high-score category patients (HR = 4.08, 95% CI: 2.19–7.61, *p* < 0.01) showed a significantly higher risk of all-cause mortality during follow-up than the low-score category patients. The high-score category patients showed a significantly higher risk of all-cause mortality than intermediate-score category patients (HR = 1.71, 95% CI: 1.01–3.15, *p* = 0.04). In the ECMO group, the intermediate-score (HR = 1.47, 95% CI: 1.06–2.03, *p* = 0.02) and high-score category patients (HR = 1.60, 95% CI: 1.04–2.46, *p* = 0.03) showed a higher risk of all-cause mortality than the low-score category patients. However, there was no difference in the risk of all-cause mortality (HR = 1.21, 95% CI: 0.81–1.81, *p* = 0.33) between the intermediate-score and high-score category patients in the ECMO group.

### 3.4. Discrimination Performance o thef IABP-SHOCK II Score for Non-Mechanical Support/IABPs Compared with ECMOs

**Figure 4:**
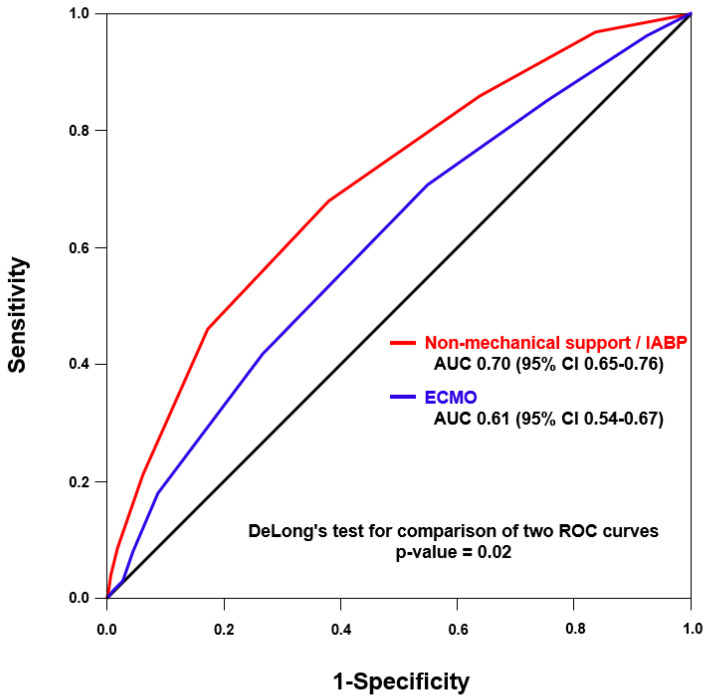
Receiver operating characteristic curves for IABP-SHOCK II score prediction of all-cause mortality between the non-mechanical support/IABP and ECMO groups.

Based on the ROC analyses, the optimal cut-off levels for the IABP-SHOCK II score to predict all-cause mortality were 3.5 (AUC = 0.70, 95% CI: 0.65–0.76) for an aggregation of the non-mechanical support and IABP groups and 2.5 points (AUC = 0.61, 95% CI: 0.54–0.67) for the ECMO group. According to ROC curves, the IABP-SHOCK II score showed a better predictive performance for in-hospital mortality in the non-mechanical support/IABP group than the ECMO group (*p*-value for comparison = 0.02; Figure 4).

## 4. Discussion

In this study, using a large-scale, multicenter dedicated CS registry, we evaluated the prognostic impact of the IABP-SHOCK II score in ACS patients complicated by CS managed according to contemporary clinical practice. Our major findings were: (1) the mortality in patients with ACS complicated by CS remained high, and high-risk patients in the ECMO group had the highest mortality risk during follow-up compared with patients treated with IABPs or non-mechanical support; (2) the IABP-SHOCK II score yielded a better risk stratification for mortality in patients of same configuration in the IABP-SHOCK II trial, compared with patients with selected severe forms of CS requiring ECMOs; and (3) the discrimination performance of the IABP-SHOCK II score in the ECMO group was modest compared with the IABP and non-mechanical support groups.

In a high-severity scenario such as ACS with CS, immediate risk stratification offers important prognostic information and may guide treatment choice, such as the use of MCS devices [15,16,17,18]. Recently, a more advanced temporary mechanical hemodynamic support device was introduced as a CS management option. Even though IABPs are still the most widely used MCS devices for CS, powerful MCS devices such as Impella and ECMOs have been increasingly used in clinical practice [19]. The IABP-SHOCK II score, derived from the IABP-SHOCK II trial, is based on a simple scoring calculation and easily applicable to clinical practice. This score is based on a homogeneous patient population with acute myocardial infarction (AMI)-related CS who are underwent early revascularization, and the scoring system performed well for predicting the short-term mortality risk [11]. However, IABP-SHOCK II scores were not validated in patients requiring advanced MCS devices. A recently published study found no significant difference in the observed mortality between patients with intermediate and high IABP-SHOCK II scores but substantial differences between the observed mortality and predicted mortality in patients requiring Impella [12].

In this study, we applied the IABP-SHOCK II score to patients with ACS complicated by CS from the RESCUE registry, a large multicenter clinical registry of CS patients. More than 50% of enrolled patients received either IABPs or ECMOs, representing contemporary management of high-severity CS. The IABP-SHOCK II score showed a good predictive performance in patients treated with non-mechanical or IABP support; however, there was no difference in the risk of all-cause mortality between intermediate-risk and high-risk patients in the ECMO group, and the discrimination performance of the IABP-SHOCK II score was modest compared with its performance in the non-mechanical support and IABP groups. These previous and current findings suggest that the discrimination ability of the IABP-SHOCK II score for mortality prediction may be unsatisfactory in situations requiring advanced mechanical circulatory support.

A few studies have evaluated predictors of worse outcomes and have suggested using predictive CS mortality models for cases that required advanced MCS devices. The Survival After Veno-arterial-ECMO (SAVE) score, which is based on an international cohort of cardiogenic shock patients receiving ECMOs, includes 12 pre-ECMO variables [20]. However, the cohort of patients used to evaluate the SAVE score system primarily included patients who received ECMOs regardless of the CS etiology. For patients with AMI-related refractory CS, Muller et al. proposed using the ENCOURAGE (prEdictioN of Cardiogenic shock OUtcome foR AMI patients salvaGed by VA-ECMO) risk score, which is a combination of seven simple variables that are readily available during pre-ECMO implantation [21]. In another study, the AMI-ECMO scoring system, which was evaluated for AMI patients treated with VA-ECMOs from a single-center registry, was introduced for mortality prediction using a variable for pre-ECMO implantation combined with angiographic data [22]. Currently, although there are two risk models focused on AMI patients treated with ECMOs, these models consisted of only pre-ECMO variables. In practice, bleeding, hemolysis, sepsis, and procedure-related complications act as triggers for clinical deterioration and mortality determinants in a substantial number of patients treated with advanced MCS.

Recently, the Society for Cardiovascular Angiography and Intervention (SCAI) proposed a simple, clinically applicable CS staging system [23]. The SCAI shock stage classified patients into five stages (A–E) that reflect progressively increasing CS illness severity that could shift to higher or lower shock stages according to dynamic clinical symptomatology and hemodynamics. The application of the SCAI shock classification to retrospective CS registry cases showed that a higher stage was associated with an increased risk of in-hospital mortality [24,25]. In particular, patients in SCAI stage E experienced circulatory collapse and were supported by multiple simultaneous acute interventions, including ECMO-facilitated resuscitation [26,27]. The very recently published randomized Extracorporeal Life Support in Cardiogenic Shock (ECLS-SHOCK) trial include predominantly AMI patients with SCAI shock stages C through E, and one-third of patient were SCAI shock stage E. However, the ECLS-SHOCK trial provided neutral results in terms of the primary endpoint (all-cause mortality at 30 days), as well as secondary endpoints, between the ECLS and control groups and additionally demonstrated an increased risk of ischemic vascular complications and bleeding in the ECLS group [28].

In the management of ACS combined with CS, there are still unsolved and complex issues, such as the optimal revascularization strategies of multivessel CAD in infarct-related CS requiring MCS and the optimal timing of revascularization. The CULPRIT-SHOCK (Culprit Lesion Only PCI Versus Multi-vessel PCI in Cardiogenic Shock) trial demonstrated that culprit-only percutaneous coronary intervention (PCI) was superior to immediate multivessel PCI in terms of short-term mortality [29]; however, when and how to employ non-culprit lesion revascularization strategy in refractory CS requiring MCS remains controversial [30,31]. Future randomized trials are warranted to determine the definitive role of a tailored complete revascularization (whether anatomical or functional) in an AMI with CS scenario and the timing of complete revascularization during MCS-supported index PCI or in a staged PCI after the acute phase [32,33]. Although the role of emergency coronary artery bypass grafting is currently underrated, surgical revascularization still represents an important treatment option in selective scenarios, and the research on surgical revascularization of refractory CS requiring MCS is lacking [34,35,36].

The patients with AMI and CS who require MCS have heterogenous risk factors not only at initial presentation but also during management. In the management of CS with ECMO support, ECMO-related complications are not uncommon, and it is well known that ECMO complications have a major negative impact on clinical outcomes. In this registry, ECMO-related complications occurred in 75 (25.3%) patients of CS requiring ECMOs, and all-cause mortality was increased with the presence of ECMO complications in the ECMO group (Supplementary Table S1). The occurrence of ECMO complications may attenuate the discrimination ability of the IABP-SHOCK II score for mortality prediction in CS patients requiring ECMOs. Taken together, this study and previous studies suggest that new insights into risk stratification according to CS severity are needed. Furthermore, future risk stratification should not be uniform at initial presentation and should be approximately individualized and reevaluated according to changes in clinical manifestations and MCS-related variables, particularly in CS patients treated with advanced MCS.

### Limitations

This study has several limitations. First, its design is non-randomized and observational, potentially affecting our results due to selection bias and confounding factors. Second, the choice of CS treatment, including the type and amount of fluid or administered vasopressor/inotrope and the type and timing of MCS use, was at the physician’s discretion. However, the coronary intervention was based on the guidelines from the Korean Circulation Society. Third, this registry did not include all mechanical circulatory support cases, because Impella is not currently available in Korea. Fourth, due to the retrospective and prospective nature of our registry, we could not thoroughly identify the detailed cause of mortality, possibly limiting our results.

## 5. Conclusions

In ACS-complicated CS patients, risk stratification using the IABP-SHOCK II score is generally useful for predicting mortality; however, its prognostic value may be unsatisfactory in patients requiring advanced MCS. Therefore, further studies are necessary for developing a risk stratification system that reflects the prognosis in CS patients in various clinical severity situations.

## Figures and Tables

**Table 1:** Baseline characteristics.

	Non-Mechanical Support	IABP	ECMO	*p*-Value
	(n = 340)	(n = 228)	(n = 297)	
Demographic characteristics				
Age (years)	66.7 ± 13.0	70.7 ± 11.1	65.6 ± 12.1	<0.01
Age > 73 years	112 (32.9)	106 (46.5)	87 (29.3)	<0.01
Male	246 (72.4)	159 (69.7)	225 (75.8)	0.29
Body mass index (kg/m^2^)	23.6 ± 3.3	23.8 ± 3.6	23.7 ± 3.1	0.85
Vital sign at shock date				
systolic blood pressure (mmHg)	76.6 ± 18.5	88.3 ± 33.3	73.8 ± 25.8	<0.01
diastolic blood pressure (mmHg)	48.8 ± 12.7	54.6 ± 20.3	49.3 ± 17.3	<0.01
systolic blood pressure < 70 mmHg	102 (30.0)	61 (26.8)	143 (48.1)	<0.01
mean blood pressure < 50 mmHg	83 (24.4)	51 (22.4)	98 (33.0)	0.01
heart rate (beat/min)	74.9 ± 27.0	88.2 ± 27.4	85.9 ± 30.9	<0.01
Presentation with STEMI	218 (64.1)	141 (61.8)	193 (65.0)	0.75
Presentation with non-ST elevation ACS	122 (35.9)	87 (38.2)	104 (35.0)	0.75
Medical characteristics				
Hypertension	185 (54.4)	136 (59.6)	163 (54.9)	0.42
Diabetes	101 (29.7)	97 (42.5)	127 (42.8)	<0.01
Dyslipidemia	98 (28.8)	83 (36.4)	76 (25.6)	0.02
Previous myocardial infarction	31 (9.1)	39 (17.1)	46 (15.5)	0.01
Previous PCI	37 (10.9)	32 (14.0)	53 (17.8)	0.04
Previous bypass graft surgery	5 (1.5)	3 (1.3)	9 (3.0)	0.26
Previous stroke	24 (7.1)	26 (11.4)	26 (8.8)	0.20
Previous peripheral artery disease	10 (2.9)	10 (4.4)	10 (3.4)	0.64
Laboratory characteristics				
Creatinine (mg/dL)	1.3 ± 1.1	1.6 ± 1.8	1.6 ± 1.5	0.06
Creatinine > 1.5 mg/dL	72 (21.2)	65 (28.5)	100 (33.7)	<0.01
Total bilirubin (mg/dL)	0.8 ± 0.7	0.8 ± 0.7	0.8 ± 1.5	0.58
Total bilirubin > 2.0 mg/dL	19 (5.6)	14 (6.1)	18 (6.1)	0.95
Glucose (mg/dL)	208.1 ± 113.4	220.8 ± 105.1	262.0 ± 127.6	<0.01
Glucose > 191 mg/dL	140 (41.2)	118 (51.8)	194 (65.3)	<0.01
Lactate (mmol/L)	5.7 ± 3.7	6.3 ± 3.8	7.94 ± 4.2	<0.01
Lactate > 5 mmol/L	160 (47.1)	135 (59.2)	207 (69.7)	<0.01

Values are mean ± standard deviation or n (%); ACS = acute coronary syndrome, IABP = intra-aortic balloon pump, ECMO = extracorporeal membrane oxygenator, STEMI = ST-segment elevation myocardial infarction, PCI = percutaneous coronary intervention.

**Table 2:** Angiographic findings and in-hospital management.

	Non-Mechanical Support	IABP	ECMO	*p*-Value
	(n = 340)	(n = 228)	(n = 297)	
Angiographical characteristic				
LM or proximal LAD involvement	239 (70.3)	203 (89.0)	264 (88.9)	<0.01
Multi-vessel disease	215 (63.2)	174 (76.3)	250 (84.2)	<0.01
Coronary artery bypass surgery	5 (1.5)	15 (6.6)	20 (6.7)	<0.01
Unsuccessful or unexecuted revascularization	9 (2.6)	17 (7.5)	22 (7.4)	0.01
In-hospital management				
Number of inotropes or vasopressors used				
1	210 (61.8)	101 (44.3)	74 (24.9)	<0.01
≥2	130 (38.2)	127 (55.7)	223 (75.1)	<0.01
Continuous renal replacement therapy	24 (7.1)	35 (15.4)	120 (40.4)	<0.01
Use of mechanical ventilation	103 (30.3)	131 (57.5)	265 (89.2)	<0.01

Values are n (%); IABP = intra-aortic balloon pump, ECMO = extracorporeal membrane oxygenator, LM = left main artery, LAD = left anterior descending artery.

**Table 3:** Hazards for all-cause mortality overall and in each management strategy group according to IABP-SHOCK II score.

Overall treatment (n = 865)		Low (n = 420)	Intermediate (n = 349)	**High (n = 96)**
	All-cause mortality	121 (28.8)	183 (52.4)	67 (69.8)
	HR (95% CI)	Reference	1.81 (1.55–2.11)	2.16 (1.72–2.72)
	*p*-value	-	<0.01	<0.01
Non-mechanical support (n = 340)		Low (n = 213)	Intermediate (n = 102)	High (n = 25)
	All-cause mortality	36 (16.9)	34 (33.3)	16 (64.0)
	HR (95% CI)	Reference	2.06 (1.27–3.32)	5.33 (2.91–9.75)
	*p*-value	-	<0.01	<0.01
IABP (n = 228)		Low (n = 101)	Intermediate (n = 98)	High (n = 29)
	All-cause mortality	25 (24.8)	43 (43.9)	18 (62.1)
	HR (95% CI)	Reference	2.05 (1.24–3.36)	4.08 (2.19–7.61)
	*p*-value	-	<0.01	<0.01
ECMO (n = 297)		Low (n = 106)	Intermediate (n = 149)	High (n = 42)
	All-cause mortality	60 (56.6)	106 (71.1)	33 (78.6)
	HR (95% CI)	Reference	1.47 (1.06–2.03)	1.60 (1.04–2.46)
	*p*-value	-	0.02	0.03

HR = hazard ratio, CI = confidence interval, IABP = intra-aortic balloon pump, ECMO = extracorporeal membrane oxygenator.

## Data Availability

The datasets used during this research are not publicly available because of privacy and ethical restrictions. However, they are available from the corresponding author on reasonable request.

## Supplementary Materials

The following supporting information can be downloaded at https://www.mdpi.com/article/10.3390/medicina60010183/s1, Table S1: All-cause mortality, ECMO complications, and all-cause mortality with ECMO complications in the ECMO group according to each IABP-SHOCK II score category.
