# Supplementary material for: Differential Prognostic Impact of IABP-SHOCK II Scores According to Treatment Strategy in Cardiogenic Shock Complicating Acute Coronary Syndrome: From the RESCUE Registry
Source: Medicina (Kaunas). 2024 Jan 20;60(1):183. doi: 10.3390/medicina60010183 (PMC10818598; doi:10.3390/medicina60010183)
Supplement: Supplementary file 1 [file medicina-60-00183-s001.zip › medicina-2790563-supplementary.pdf]

Supplementary Table S1. All-cause mortality, ECMO complication and all-cause mortality with ECMO complication in ECMO group according to each IABP-SHOCK II score category

	ECMO group total (n=297)	Low (n=106)	Intermediate (n=149)	High (n=42)	P-value
**All-cause mortality**	199 (67.0%)	60 (56.6%)	106 (71.1%)	33 (78.6%)	0.01
**ECMO complication**	75 (25.3%)	18 (16.9%)	45 (30.2%)	12 (28.6%)	0.04
ECMO site bleeding	39 (13.1)	9 (8.5)	24 (16.1)	6 (14.3)	0.03
GI bleeding	16 (5.4)	3 (2.8)	10 (6.7)	3 (7.1)	0.08
Limb ischemia	20 (6.7)	4 (3.8)	14 (9.4)	2 (4.7)	0.05
Sepsis	11 (3.7)	1 (0.9)	8 (5.4)	2 (4.7)	0.54
Hemorrhagic/ischemic stroke	13 (4.3)	6 (5.6)	5 (3.4)	2 (4.7)	0.82
**ECMO complication with all-cause mortality**	57 (19.1%)	11 (10.4%)	34 (22.8%)	12 (28.6%)	0.01
